# Error in Author Affiliations

**DOI:** 10.1001/jamanetworkopen.2026.33551

**Published:** 2026-08-17

In the Original Investigation titled “At-Home Transvaginal Pelvic Ultrasonography and Image Quality in Premenopausal Women: A Nonrandomized Clinical Trial,”^1^ published July 6, 2026, there was an error in the Author Affiliations. Tina Islam, MD, should have been affiliated with Academy Hill Teleradiology LLC, Boston, Massachusetts.^1^
